# Low expression of BMPRIB indicates poor prognosis of breast cancer and is insensitive to taxane-anthracycline chemotherapy

**DOI:** 10.18632/oncotarget.6613

**Published:** 2015-12-14

**Authors:** Kun Dai, Fengxia Qin, Huikun Zhang, Xiaoli Liu, Caixia Guo, Ming Zhang, Feng Gu, Fu Li, Yongjie Ma

**Affiliations:** ^1^ Department of Breast Cancer Pathology and Research Laboratory, Key Laboratory of Breast Cancer Prevention and Therapy (Ministry of Education), Tianjin Medical University Cancer Institute and Hospital, National Clinical Research Center for Cancer, Tianjin, China; ^2^ Department of Tumor Cell Biology, Key Laboratory of Cancer Prevention and Therapy of Tianjin, Tianjin Medical University Cancer Institute and Hospital, National Clinical Research Center for Cancer, Tianjin, China; ^3^ CAS Key Laboratory of Genomic and Precision Medicine, Beijing Institute of Genomics, Chinese Academy of Sciences, Beijing, China; ^4^ Department of Epidemiology and Biostatistics, University of Georgia, Athens, GA, U.S.A

**Keywords:** BMPRIB, breast cancer, bone metastasis, TE chemotherapy, prognosis

## Abstract

Bone morphogenetic protein receptor type IB (BMPRIB) is one osteogenesis factor, which function in breast cancer has been rarely explored until recently. In the clinical study presented here, involving a cohort of 368 invasive ductal carcinoma (IDC) patients, we identified that patients with low expression of BMPRIB exhibited poor prognosis, especially in the luminal B subtype. We also provided the first piece of evidence that low level of BMPRIB was a promoting factor for breast cancer patients to develop bone metastasis, but not lung, liver or brain. The first of its kind, we reported that patients with high expression of BMPRIB exhibited favorable prognosis by a retrospective analysis consisting of 168 patients treated with TE (taxane and anthracycline) regimens. And the patients with high expression of BMPRIB were more sensitive to TE regimens in the detection of 32 paired pre-neoadjuvant and post-neoadjuvant specimens. Overall, our study concluded that low expression of BMPRIB indicated poor prognosis of breast cancer and was insensitive to taxane-anthracycline chemotherapy. Our findings also lay a foundation to help clinicians improve identification of patients for TE regimens by BMPRIB in the era of precision medicine.

## INTRODUCTION

Breast cancer is a common cancer in women and a major cause of cancer death among women of all races and populations, due to the development of secondary tumors in vital organs [1]. First metastatic lesions are found at the highest frequency in bone (83%) whereas liver and lung are usually affected to a lesser extent (27%) [2–3].

Bone morphogenetic protein receptor type IB (BMPRIB) as a member of BMPRs, could lead to the activation of intracellular signaling pathways and inhibited cell proliferation, migration, and invasiveness [4–7]. It has been reported that BMPRIB expression was higher in the normal, benign specimens and well-differentiated tumors compared with poorly-differentiated tumors in human prostate cancer, glioma, and ovarian cancer [8–12].

Reports of BMPRIB in breast cancer are few and contradictory [5, 13]. It was reported high expression of BMPRIB indicated a poor prognosis in estrogen receptor-positive breast cancer patients [13]. However, Bokobza et al.'s study presented an opposite conclusion in prognosis [5]. Such confused reports were probably due to small samples or unscientific division. Until present, there is still no comprehensive description which is capable of pointing out the accurate relationship between BMPRIB and breast cancer, particularly distant metastasis.

In our present clinicopathologic study, we presented immunohistochemistry analysis of BMPRIB expression in a large population of 368 cases of invasive ductal carcinoma (IDC), which covered all molecular subtypes. We confirmed that low expression of BMPRIB predicted poor prognosis in IDC patients. In addition, we found that IDC patients with low expression of BMPRIB exhibited bone-specific metastasis, but not lung, liver or brain. Furthermore, we reported firstly that high level of BMPRIB expression predicted a favorable prognosis in patients treated with TE (taxane and anthracycline) regimens. Patients with high expression of BMPRIB were more sensitive to TE regimens which was validated by detecting 32 paired pre-neoadjuvant and post-neoadjuvant specimens. Overall, our findings demonstrated that low expression of BMPRIB indicated poor prognosis of breast cancer and was insensitive to taxane-anthracycline chemotherapy.

## RESULTS

### Low expression of BMPRIB promoted breast cancer progression

**Figure 1:**
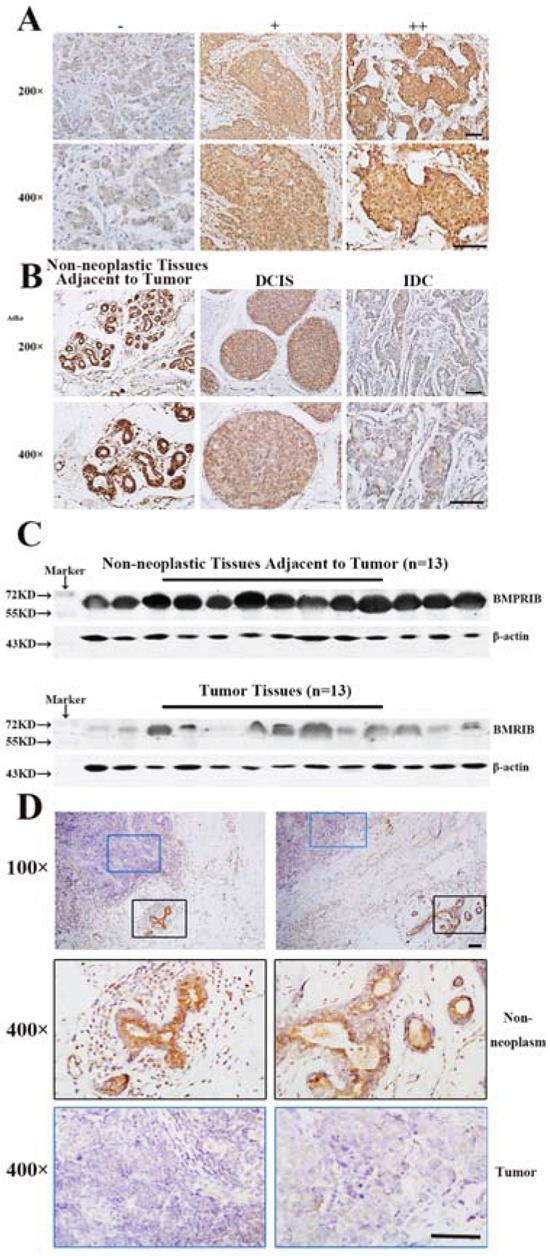
Low expression of BMPRIB promoted breast cancer progression **A.** Varying degree staining intensity of BMPRIB protein in invasive ductal carcinoma specimens: (−): no or low staining; (+): moderate staining; (++): strong staining. **B.** Immunohistochemistry of BMPRIB in clinical specimens of non-neoplastic breast tissues adjacent to tumor, ductal carcinoma in situ (DCIS) and invasive ductal carcinoma (IDC) (magnification 200× and 400×). **C.** Western blot analysis of BMPRIB expression in breast tumor specimens (n = 13) and non-neoplastic breast tissues adjacent to tumor (n = 13). β-actin was used as a loading control. **D.** BMPRIB expression was detected in two typical sections (left part and right part) respectively, both of which contained non-neoplastic tissues and tumor tissues. Blue rectangle represented tumors and black rectangle represented non-neoplastic tissues adjacent to tumor. (Bar = 100μm)

Expression of BMPRIB was detected in 52 cases of non-neoplastic tissues adjacent to tumor, 40 cases of DCIS and 368 cases of IDC. The immunohistochemical staining of BMPRIB was assessed and the intensity of staining was shown in representative images as Figure 1A. In breast tissues, BMPRIB was mainly located in the cytoplasm of epithelial cells of the mammary gland ducts. We found the expression of BMPRIB was gradually down-regulated from non-neoplastic tissues adjacent to tumor, to DCIS and to IDC (Figure 1B). 26.9% (14/52) of non-neoplastic tissues adjacent to tumor, 55.0% (22/40) of DCIS and 60.3% (222/368) of IDC tissue specimens showed low expression of BMPRIB (*r_s_* = −0.184, *P* < 0.001) (Table 1). Furthermore, Western blot analyses were employed to show the protein expression of BMPRIB in frozen IDC specimens (13 cases) and non-neoplastic breast specimens (13 cases), respectively. We confirmed that expression of BMPRIB was lower in IDC specimens compared with non-neoplastic breast specimens (Figure 1C). We also examined BMPRIB expression in paraffin sections and each section contained both non-neoplasm and tumor. The typical immunohistochemistry images of BMPRIB expression in two cases were shown in Figure 1D.

**Table 1:** BMPRIB expression in different breast tissue specimens

Histological type	n	BMPRIB score, n (%)		rs	P value
		Low (0-140)	High (141-200)		
Non-neoplastic Tissues	52	14 (26.9)	38 (73.1)		
DCIS	40	22 (55.0)	18 (45.0)	−0.184	<0.001**
IDC	368	222 (60.3)	146 (39.7)		

Non-neoplastic Tissues: Non-neoplastic Tissues Adjacent to Tumor

DCIS: ductal carcinoma in situ

** *P* value was calculated by Spearman's Rank-Correlation test.

BMPRIB expression was negatively correlated with tumor size (*r_s_* = −0.190, *P* < 0.001), cTNM stage (*r_s_* = −0.126, *P* = 0.016), lymph node metastasis (*r_s_* = −0.202, *P* < 0.001) and distant metastasis (*r_s_* = −0.148, *P* = 0.004) but positively associated with the expression of PR (*r_s_* = 0.210, *P* < 0.001) of breast cancer. No significant associations were identified between the expression of BMPRIB and patients' age (*r_s_* = −0.056, *P* = 0.286), histological grade (*r_s_* = 0.038, *P* = 0.472), ER (*r_s_* = 0.064, *P* = 0.223), or HER2 (*r_s_* = 0.016, *P* = 0.758) (Table 2).

**Table 2:** BMPRIB expression and pathological features of IDC

Pathological features	n	BMPRIB score, n (%)		rs	P value
		Low (0-140)	High (141-200)		
Age, year				−0.056	0.286
50	189	109 (57.7)	80 (42.3)		
≥50	179	113 (63.1)	66 (36.9)		
Tumor size, cm				−0.190	<0.001**
≤2	79	34 (43.0)	45 (57.0)		
2-5	230	145 (63.0)	85 (37.0)		
>5	59	43 (72.9)	16 (27.1)		
cTNM stage☨				−0.126	0.016*
I	60	28 (46.7)	32 (53.3)		
II	237	146 (61.6)	91 (38.4)		
III-IV	69	47 (68.1)	22 (31.9)		
Histological grade☨				0.038	0.472
I	10	5 (50.0)	5 (50.0)		
II	272	164 (60.3)	108 (39.7)		
III	64	37 (57.8)	27 (42.2)		
LN metastasis				−0.202	<0.001**
NO	142	68 (47.9)	74 (52.1)		
YES	226	154 (68.1)	72 (31.9)		
Distant metastasis				−0.148	0.004**
NO	317	182 (57.4)	135 (42.6)		
YES	51	40 (78.4)	11 (21.6)		
Estrogen receptor				0.064	0.223
Negative	122	79 (64.8)	43 (35.2)		
Positive	246	143 (58.1)	103 (41.9)		
Progesterone receptor				0.210	0.001**
Negative	109	83 (76.1)	26 (23.9)		
Positive	259	139 (53.7)	120 (46.3)		
HER2/neu				0.016	0.758
Negative	291	180 (61.9)	111 (38.1)		
Positive	77	42 (54.5)	35 (45.5)		

☨ Some missing data.

LN metastasis: lymph node metastasis

*P* values were calculated by Spearman's Rank-Correlation test.

### Low expression of BMPRIB in IDC patients indicated worse prognosis

**Figure 2:**
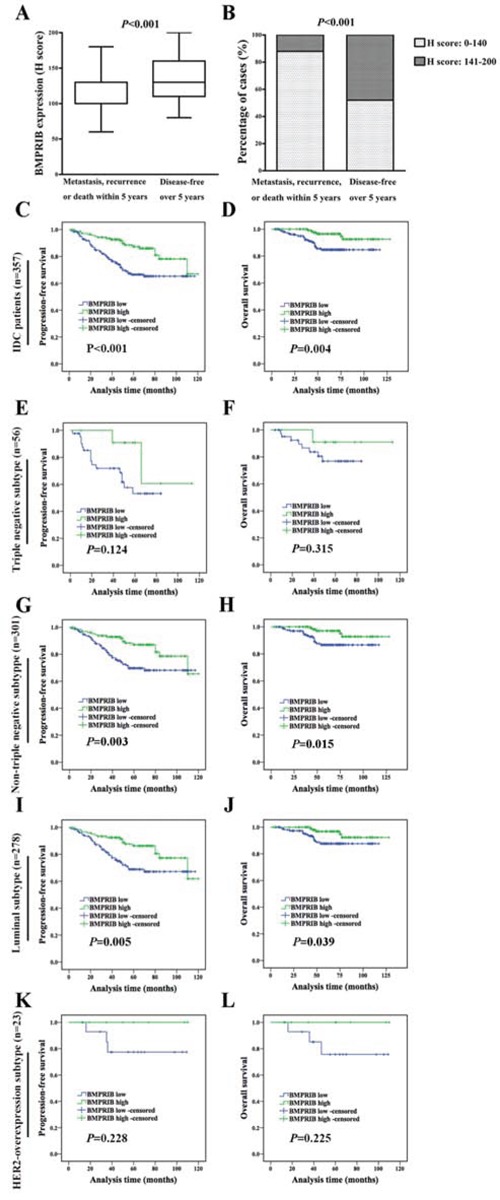
Low expression of BMPRIB in IDC patients indicated worse prognosis **A.** BMPRIB expression in patients who developed metastasis, recurrence or death within 5 years was lower than that in those who were disease-free over 5 years (Mann-Whitney U test, *P* < 0.001). **B.** Percentage of cases with low and high expression of BMPRIB with regard to patients' prognosis. 87.5% (42/48) of patients who developed metastasis, recurrence or death within 5 years showed lower expression of BMPRIB, while 50.7% (74/144) of patients were disease-free over 5 years (χ^2^ test, *P* < 0.001). **C** and **D.** Progression-free survival (PFS) and overall survival (OS) curves of IDC patients (n = 357) with BMPRIB expression, respectively (log-rank test). **E** and **F.** PFS and OS curves of triple negative subtype patients (n = 56) with BMPRIB expression, respectively. **G** and **H.** PFS and OS curves of non-triple negative subtype patients (n = 301) with BMPRIB expression, respectively. **I** and **J.** PFS and OS curves of luminal subtype patients (n = 278) with BMPRIB expression, respectively. **K** and **L.** PFS and OS curves of HER2-overexpression subtype patients (n = 23) with BMPRIB expression, respectively.

In order to explore the role of BMPRIB in breast cancer prognosis, we analyzed 357 IDC patients with complete clinical follow-up. We found BMPRIB expression in patients with metastasis, recurrence or death within 5 years (H score: 60.0 to 180.0, median: 100.0) was lower than those who were disease-free over 5 years (H score: 80.0 to 200.0, median: 130.0) (*P* < 0.001, Figure 2A). Cases with low BMPRIB expression were 87.5% (42/48) and 51.4% (74/144) in metastasis, recurrence or death within 5 years group and disease-free over 5 years group (*P* < 0.001) (Figure 2B). Both PFS and OS in IDC patients with low expression of BMPRIB were shorter than that of patients with high expression of BMPRIB (Figure 2C, 2D).

**Figure 3:**
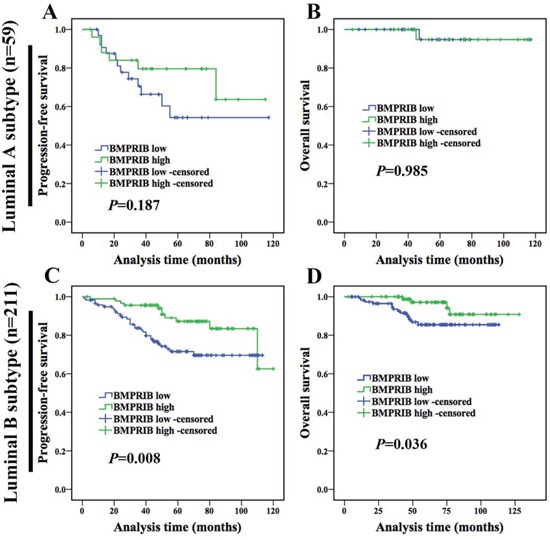
Low expression of BMPRIB in luminal B subtype patients indicated worse prognosis **A** and **B** were PFS and OS curves of luminal A subtype patients (n = 59) with BMPRIB expression, respectively. **C** and **D** were PFS and OS curves of luminal B subtype patients (n = 211) with BMPRIB expression, respectively.

Kaplan-Meier analysis of triple negative subtype (56 cases) showed that the expression of BMPRIB was not correlated with PFS (*P* = 0.124, Figure 2E) or OS (*P* = 0.315, Figure 2F). Then we investigated the correlation between BMPRIB and prognosis in non-triple negative subtype patients (n = 301). Kaplan-Meier analysis showed that low expression of BMPRIB indicated shorter PFS (*P* = 0.003, Figure 2G) and OS (*P* = 0.015, Figure 2H). In the following, we divided non-triple negative cases into two groups: luminal subtype (278 cases) and HER2-overexpression subtype (23 cases). We found no correlation between BMPRIB and PFS (*P* = 0.228, Figure 2K) or OS (*P* = 0.225, Figure 2L) in HER2-overexpression subtype. However, Kaplan-Meier analysis showed that low expression of BMPRIB indicated shorter PFS (*P* = 0.005, Figure 2I) and OS (*P* = 0.039, Figure 2J) in luminal subtype, especially in luminal B subtype (PFS: *P* = 0.008; OS: *P* = 0.036; Figure 3).

### Low level of BMPRIB was a promoting factor for breast cancer patients to develop bone metastasis

In the present study, we found a negative correlation between expression of BMPRIB and bone metastasis in 357 IDC patients (*r_s_* = −0.119, *P* = 0.024). No correlations were identified between BMPRIB expression and distant metastasis to other organs, such as lung (*r_s_* = −0.056, *P* = 0.289), liver (*r_s_* = −0.036, *P* = 0.500) and brain (*r_s_* = −0.048, *P* = 0.364, only 5 cases, data not shown) (Table 3).

**Table 3:** Relationship between BMPRIB expression and distant metastasis in invasive breast cancer

Distant metastasis	n	BMPRIB score, n (%)		rs	P value
		Low (0-140)	High (141-200)		
Bone metastasis				−0.119	0.024*
NO	318	185 (58.2)	133 (41.8)		
Yes	39	30 (76.9)	9 (23.1)		
Lung metastasis				−0.056	0.289
No	345	206 (59.7)	139 (40.3)		
Yes	12	9 (75.0)	3 (25.0)		
Liver metastasis				−0.036	0.500
No	344	206 (59.9)	138 (40.1)		
Yes	13	9 (69.2)	4 (30.8)		

*P* value was calculated by Spearman's Rank-Correlation test.

**Figure 4:**
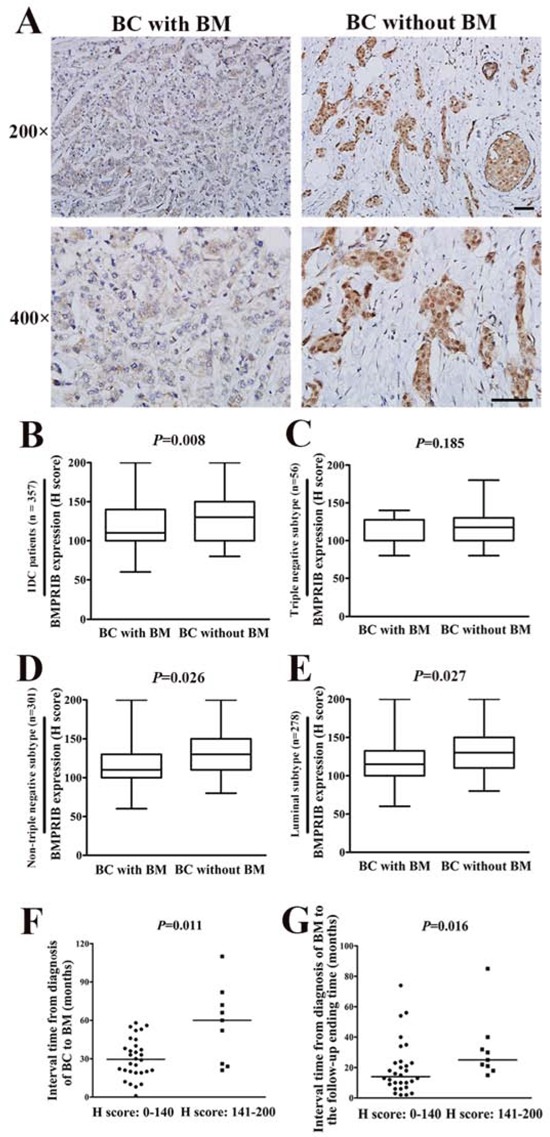
Low level of BMPRIB was a promoting factor for breast cancer (BC) patients to develop bone metastasis (BM) **A.** Representative immunohistochemical images of BMPRIB expression in primary tumor specimens of BC with BM and BC without BM, respectively (magnification 200× and 400×). (Bar = 100μm) **B.** BMPRIB expression in BC patients who developed BM was lower than that in those patients without BM (*P* = 0.008). **C.** Comparison of BMPRIB expression in BC with BM and BC without BM in triple negative subtype IDC patients (*P* = 0.185). **D.** Comparison of BMPRIB expression in BC with BM and BC without BM in non-triple negative subtype IDC patients (*P* = 0.026). **E.** Comparison of BMPRIB expression in BC with BM and BC without BM in luminal subtype IDC patients (*P* = 0.027). **F.** BC patients with low expression of BMPRIB exhibited earlier occurrence of BM. The median interval time from diagnosis of BC to BM in patients with low expression of BMPRIB was shorter than the high BMPRIB expression group (Mann-Whitney U test, *P* = 0.011). **G.** BC patients with low expression of BMPRIB exhibited shorter survival after diagnosis of BM. The median interval time from diagnosis of BM to the follow-up ending time of patients with low expression of BMPRIB was shorter than that of high BMPRIB expression patients (*P* = 0.016). (B-G: Mann-Whitney U test).

To further investigate the relationship between BMPRIB expression and breast cancer bone metastasis, 357 IDC patients were divided into 2 groups: breast cancer with bone metastasis (n = 39) and without bone metastasis (n = 318). Expression of BMPRIB of breast cancer patients with bone metastasis was lower than that without bone metastasis (Figure 4A). The median H score of BMPRIB (110.0) of patients with bone metastasis was lower than that (130.0) without bone metastasis (*P* = 0.008, Figure 4B). In triple negative patients, BMPRIB expression in the group with bone metastasis (median, 100.0) was similar to that without bone metastasis (median, 120.0) (*P* = 0.185, Figure 4C). While in non-triple negative subtype patients, Mann-Whitney U analysis showed that patients with bone metastasis (median, 120.0) showed lower median H score of BMPRIB compared with those without bone metastasis (median, 130.0) (*P* = 0.026, Figure 4D). Furthermore, in luminal subtype, we found lower expression of BMPRIB in patients with bone metastasis compared with those without bone metastasis (*P* = 0.027, Figure 4E). We did not analyze relationship between BMPRIB expression and breast cancer bone metastasis in HER2-overexpression subtype due to only one case.

Next, we analyzed the interval time from diagnosis of breast cancer to bone metastasis and from diagnosis of bone metastasis to the follow-up ending time respectively in the 39 IDC patients with bone metastasis. IDC patients with low BMPRIB expression exhibited a trend for shorter time (median 30.0 months) to develop bone metastasis than those (median 60.0 months) with high expression (*P* = 0.011, Figure 4F). Furthermore, the interval time from diagnosis of bone metastasis to the follow-up ending time exhibited the same trend. Low BMPRIB expression has the potential for a reduced survival compared with those with high expression, and the median interval time were 13.0 months and 25.0 months, respectively (*P* = 0.016, Figure 4G). Above results indicated that high expression of BMPRIB predicted a favorable outcome in breast cancer with bone metastasis.

### High expression of BMPRIB indicated a favorable prognosis of breast cancer patients treated with TE-based regimens

It was reported that docetaxel treatment was associated with expression of BMPRIB. Docetaxel (analogs of taxane) could reduce the expression of β1-integrin and then activated BMP signaling by up-regulating SMAD1/5/8 and p38 leading to an increased expression of BMPRIB [14–16]. In the following experiments, we examined the relationship of BMPRIB and taxane combined anthracycline (TE) based therapies.

**Figure 5:**
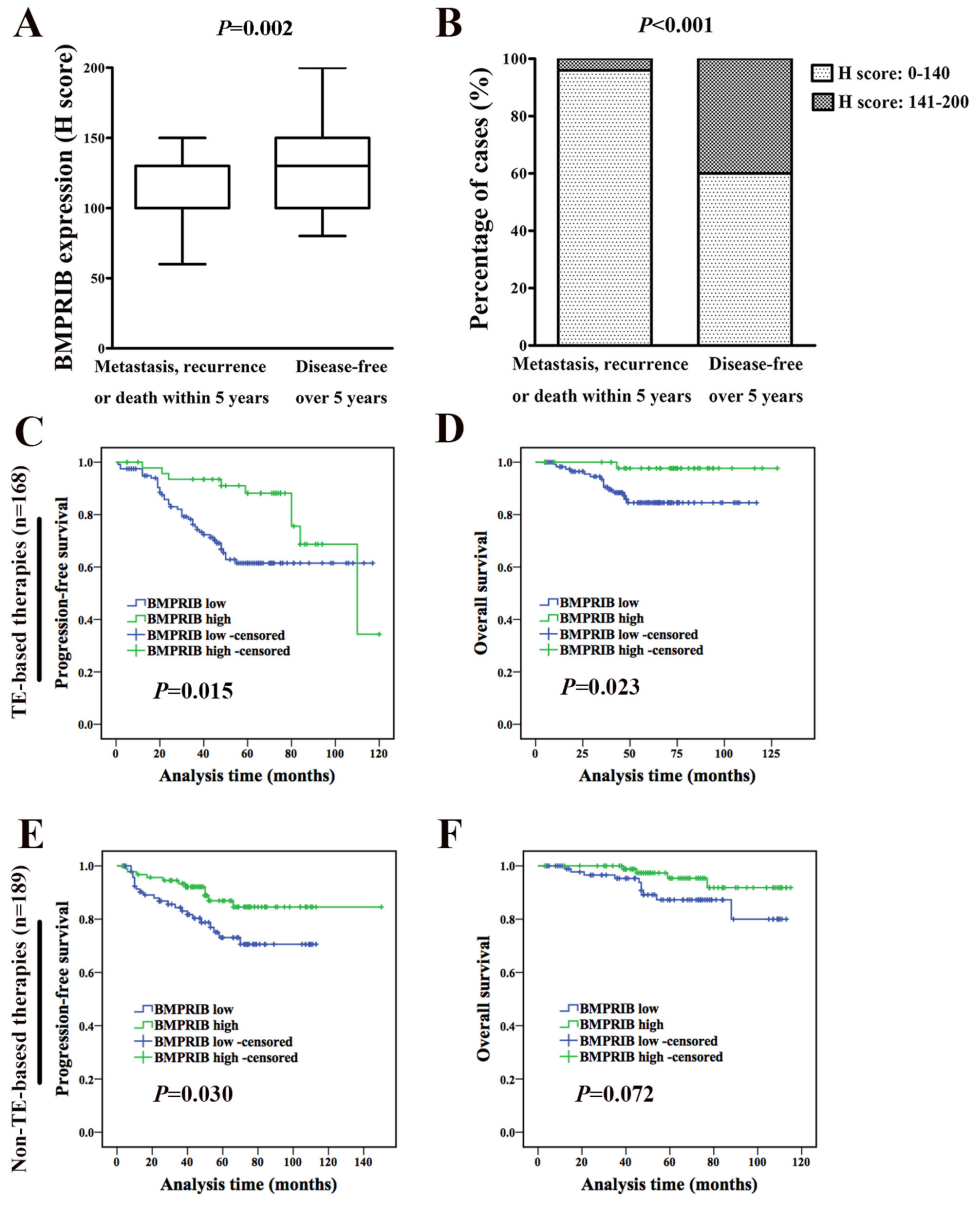
High BMPRIB expression indicated favorable prognosis in breast cancer patients treated with TE-based therapies **A.** Among 168 TE-based therapies, BMPRIB expression in patients who developed metastasis, recurrence or death within 5 years was lower than that in those patients who were disease-free over 5 years (Mann-Whitney U test, *P* = 0.002). **B.** Percentage of cases with low and high expression of BMPRIB with regard to patients treated with TE-based therapies' prognosis. 96.6% (28/29) of patients that developed metastasis, recurrence or death within 5 years showed lower BMPRIB expression compared with 59.7% (40/67) patients who were disease-free over 5 years (χ^2^ test, *P* < 0.001). **C** and **D.** Progression-free survival (PFS) and overall survival (OS) curves of IDC patients treated with TE-based therapies (n = 168) with BMPRIB expression, respectively (log-rank test). **E** and **F.** PFS and OS curves of IDC patients treated with non-TE-based therapies (n = 189) with BMPRIB expression, respectively.

We analyzed the BMPRIB expression with patient prognosis using the immunohistochemcal system in conventional TE-based regimens. Among 357 cases, 168 (47.1%) patients received TE-based chemotherapy and BMPRIB expression in patients (29 cases) who developed metastasis, recurrence or death within 5 years was lower (median, 100.0) than those (67 cases) who were disease-free over 5 years (median, 130.0) (*P* = 0.002, Figure 5A). Percentages of cases with low BMPRIB expression were 96.6% (28/29) and 59.7% (40/67) in patients who developed metastasis, recurrence or death within 5 years and patients who were disease-free over 5 years, respectively (χ^2^ = 13.303, *P* < 0.001) (Figure 5B).

Kaplan-Meier analysis showed that low expression of BMPRIB indicated shorter PFS (*P* = 0.015, Figure 5C) and OS (*P* = 0.023, Figure 5D) in patients who received TE-based therapies and followed up with a median of 60 months (5-120 months). We found no correlation between the expression of BMPRIB and OS (*P* = 0.072, Figure 5F) in patients (189 cases) who received other chemotherapy, although low expression of BMPRIB indicated a shorter PFS (P=0.030, Figure 5E). It demonstrated that high expression of BMPRIB indicated a favorable prognosis in patients who received TE-based therapies. Among 168 TE-based cases, we assessed the prognosis in different molecular subtypes. We found no correlation between BMPRIB and OS in luminal subtype (123 cases, *P* = 0.129), triple negative subtype (27 cases, *P* = 0.342) and HER2-overexpression subtype (15 cases, *P* = 0.515) (data not shown).

### Patients with low expression of BMPRIB were insensitive to TE regimens

**Figure 6:**
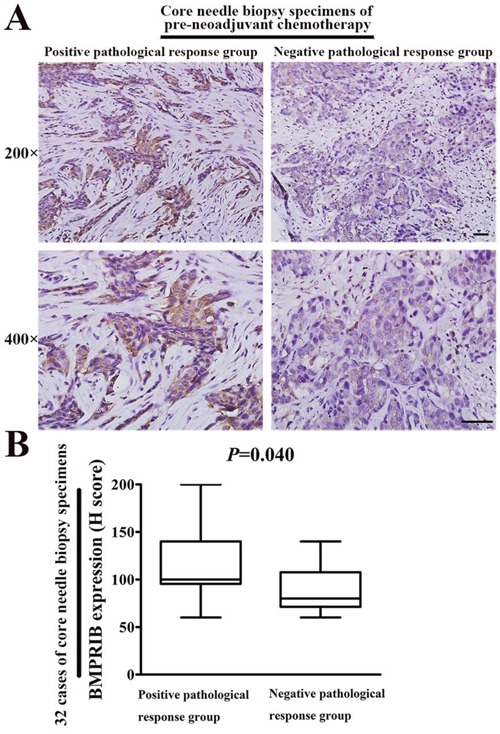
High BMPRIB expression was sensitive to TE therapy by using 32 specimens of core needle biopsy before neoadjuvant chemotherapy **A.** Representative immunohistochemical images of BMPRIB expression in both positive and negative pathological response groups (magnification 200× or 400×). **B.** BMPRIB expression was higher in positive pathological response group than negative pathological response group (Mann-Whitney U test, *P* = 0.040). (Bar = 100μm)

We also randomly selected another cohort of patients (32 cases) hospitalized during 2005 to 2009 and they were diagnosed with invasive breast cancer using core needle biopsy and then treated with TE combined chemotherapy before surgery. We divided 32 patients into positive pathological response group and negative pathological response group, on the basis of postoperative pathology. We found BMPRIB expression was higher (median, 100) in positive pathological response group (20 cases) than that (median, 80) of negative pathological response group (12 cases) in core needle biopsy specimens before TE treatment (Figure 6A, 6B; *P* = 0.040).

**Figure 7:**
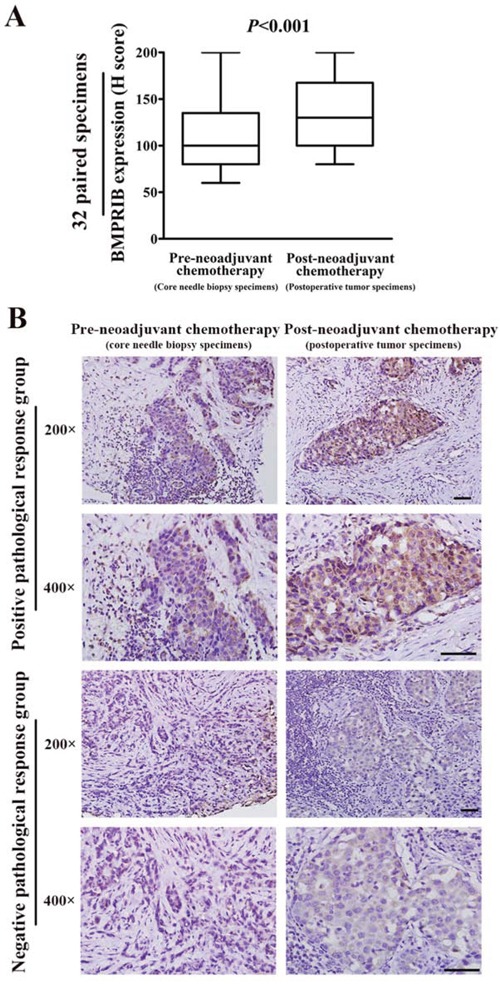
Expression of BMPRIB was up-regulated after TE neoadjuvant chemotheapy, which was mainly contributed by the positive pathological response group **A.** Expression of BMPRIB was up-regulated after TE neoadjuvant chemotheapy by 32 paired specimens of pre- and post-neoadjuvant chemotherapy (Mann-Whitney U test, *P* < 0.001). **B.** In positive pathological response group, BMPRIB expression was up-regulated in postoperative tumor specimens compared with their paired core needle biopsy specimens. Representative immunohistochemical images of BMPRIB expression in positive pathological response group were shown in upper part (magnification 200× or 400×). In negative pathological response group, there was not an obvious up-regulated BMPRIB expression in the postoperative tumor specimens compared with their paired core needle biopsy specimens. Representative immunohistochemical images of BMPRIB expression in negative pathological response group were shown in lower part (magnification 200× or 400×). (Bar = 100μm)

We found the expression of BMPRIB increased after TE neoadjuvant chemotherapy by using 32 paired specimens of pre- and post-neoadjuvant chemotherapy. Mann-Whitney U analysis showed that the median H score of BMPRIB in pre-neoadjuvant chemotherapy specimens (median: 100.0) was lower than that in post-neoadjuvant chemotherapy specimens (median: 130.0) (*P* < 0.001, Figure 7A). Percentages of cases with low BMPRIB expression were 87.5% (28/32) and 59.4% (19/32) in groups of pre-neoadjuvant chemotherapy specimens and the post-neoadjuvant chemotherapy specimens, respectively (*P* = 0.007, Table 4). In positive pathological response group, 80.0% (16/20) of pre-neoadjuvant chemotherapy specimens showed low expression of BMPRIB, which was higher than that (50.0%, 10/20) of the post-neoadjuvant chemotherapy specimens (*P* = 0.034, Table 4, Figure 7B). In negative pathological response group, there was not an obvious increase of BMPRIB expression in the post-neoadjuvant chemotherapy specimens compared with their paired pre-neoadjuvant chemotherapy specimens (*P* = 0.083, Table 4, Figure 7B).

**Table 4:** Relationship between BMPRIB expression and TE neo-adjuvant chemotherapy in 32 paired specimens

	BMPRIB score, n (%)		P value
	Low (0-140)	High (141-200)	
Total			0.007*
Pre-neoadjuvant chemotherapy	28 (87.5)	4 (12.5)	
Post-neoadjuvant chemotherapy	19 (59.4)	13 (40.6)	
Positive pathological response			0.034*
Pre-neoadjuvant chemotherapy	16 (80.0)	4 (20.0)	
Post-neoadjuvant chemotherapy	10 (50.0)	10 (50.0)	
Negative pathological response			0.083
Pre-neoadjuvant chemotherapy	12 (100.0)	0 (0.0)	
Post-neoadjuvant chemotherapy	9 (75.0)	3 (25.0)	

Statistical analysis was used Wilcoxon matched pairs signed rank test.

## DISCUSSION

Multiple factors were reported to participate in breast cancer bone metastasis [17–23]. Buijs et al. showed that high expression of BMP 7 (a member of BMP family) inhibited the formation of bone metastases in breast cancer patients [24]. Lewis et al. found loss of BMPRIB induced increased expression of cytokeratin17, which is a promoting factor of invasion [6]. In our present study, novel evidence is provided that low level of BMPRIB was a promoting factor for breast cancer patients to develop bone metastasis, but not lung, liver or brain.

TE-based therapies are part of the standard of care in first line treatment of metastatic breast cancer and their clinical use is widespread [25–26]. Actually, only about 15% patients could achieve pathologic complete response, thus a more detailed classification is necessary to screen a more suitable population to TE regimens [27]. A potential predictor of response to TE chemotherapy is urgently needed.

We reported for the first time that high BMPRIB expression was sensitive to TE-based chemotherapy by two cohorts of population. The survival analysis revealed that low expression of BMPRIB indicated a poor prognosis in 168 TE-based patients. In addition, by 32 paired pre- and post-neoadjuvant chemotherapy specimens, patients with low BMPRIB expression were insensitive to TE neoadjuvant chemotherapy. Dzietczenia et al. found that leukemia patients with a complete or partial response to anthracyclines-based chemotherapy exhibited higher expression of BMPRIB than those patients without a response to treatment which was consistent with our findings in breast cancer [28].

Overall, our study concluded that low expression of BMPRIB indicated poor prognosis of breast cancer and was insensitive to taxane-anthracycline chemotherapy. Our findings also lay a foundation to help clinicians improve identification of patients for TE regimens by BMPRIB in the era of precision medicine.

## MATERIALS AND METHODS

### Patient selection and clinical information

Paraffin-embedded specimens of 368 breast cancer patients with invasive ductal carcinoma (IDC), diagnosed between 2004 and 2009, together with 40 cases of breast ductal carcinoma in situ (DCIS) and 52 cases of non-neoplastic tissues were reviewed and randomly selected from the archives of the Department of Breast Cancer Pathology and Research Laboratory, Tianjin Medical University Cancer Institute & Hospital (Tianjin, China) for this study. The histopathology was reviewed and diagnosis in each case was confirmed independently by two pathologists according to World Health Organization (WHO) criteria. This study was reviewed and approved by the Institutional Ethic Committee of Tianjin Medical University Cancer Institute & Hospital. All the patients signed an informed consent for participation of the study and the use of their biological tissues.

368 IDC patients were women aging from 28 to 89 years (mean 51.5 years) without preoperative chemotherapy or radiation. A total of 357 cases were included for prognostic analyses, excluding cases with no follow-up data (11 cases). These patients were followed up with a median of 59 months (3-180 months). Recurrences and distant metastasis were recorded for 20 (5.4%) cases and 51 (13.9%) cases respectively, and 32 (8.7%) patients died. Among 357 cases, 278 (77.9%) patients were luminal subtype, 23 (6.4%) patients were HER2-overexpression subtype and 56 (15.7%) were triple negative subtype.

168 (47.1%) patients received TE (taxane + anthracycline) based chemotherapies, the rest (189 cases, 52.9%) were treated with other chemotherapies (not TE-based therapies) after operation. The details of patients who received non-TE based chemotherapy were in the following: 86 cases (CEF/CAF); 76 cases (CMF); 7 cases (Platinum-based chemotherapy); 4 cases (Taxane); 16 cases (unknown). (CEF: Cyclophosphamide, epirubicin and 5-fluorouracil; CAF: Cyclophosphamide, doxorubicin and 5-fluorouracil; CMF: Cyclophosphamide, methotrexate and 5-fluorouracil)

### Patients' prognostic information

Among 357 patients with prognostic analyses, there were 48 developed metastases, recurrence or death within 5 years; while 144 patients were disease-free over 5 years since diagnosis of breast cancer. Among patients who received TE-based regimens, 29 patients developed metastasis, recurrence or death within 5 years; while 67 patients who were disease-free over 5 years.

Among 357 patients with prognostic analyses, there were 51 who developed distant metastasis during the follow-up period, 39 patients developed bone metastasis, 12 patients developed lung metastasis, 13 patients developed liver metastasis, 5 patients developed brain metastasis, 2 patients developed uterus metastasis and 3 additional cases developed kidney, ovarian and thyroid metastasis, respectively. It was worth noting that multiple organic metastases were noted in 14 patients. Among those 39 IDC patients with bone metastasis, 30 patients were luminal subtype, 1 patient was HER2-overexpression subtype and 8 were triple-negative subtype.

### Information of 32 paired specimens from pre-neoadjuvant and post-neoadjuvant chemotherapy

We also randomly selected another cohort of patients (32 cases) hospitalized during October 2005 to June 2009. All 32 patients were diagnosed with invasive breast cancer by 14-gauge core needle biopsy and then had completed with preoperative neoadjuvant chemotherapy consisted of 2 to 8 cycles of TE combined chemotherapy regimen without other local or systemic treatment before surgery. Patients were women 28 to 71 years of age (mean age 52.6 years) and had no other malignant tumors or tumor history. The distribution of clinical involvement showed that all the patients had tumors >2.0 cm. These 32 paired specimens were collected from each patient's core needle biopsy specimens of primary breast tumor before neoadjuvant chemotherapy and its matching postoperative tumor tissues. All specimens were immediately fixed in 10% normal-buffered formalin and embedded in paraffin and stained for the presence of BMPRIB by immunohistochemistry.

The pathological response to neoadjuvant chemotherapy was evaluated after surgical resection of the remaining tumor and assessed according to Miller and Payne histological grading system: grade 1, no change or some alteration to individual malignant cells but no reduction in overall cellularity; grade 2, minor loss (up to 30%) of cancer cells but overall cellularity remains high; grade 3, reduction of 30% to 90% of cancer cells; grade 4, more than 90% loss of cancer cells but small clusters or widely dispersed individual cancer cells remain; grade 5, no malignant cells identifiable in sections from the site of the tumor consisting of vascular fibroblastic stroma, often containing macrophages; however, ductal carcinoma in situ (DCIS) may be present [29]. Of these 32 patients, there were 12 grade 1 responses, 9 grade 2 responses, 7 grade 3 responses and 4 grade 4 responses, but no grade 5 responses. In this study, the 32 patients were divided into two groups: one group was pathological response grade 2 to 4 which was regarded as positive and another group was pathological response grade 1 which was regarded as negative.

### Immunohistochemical staining

BMPRIB expression was examined by histochemistry techniques and S-P method. In brief, Sections (5μm thick) were dewaxed, hydrated, and heated for 2.5 min for antigen retrieval in a conventional pressure cooker by using citrate buffer, pH 6.0. Then sections were treated with 3% H_2_O_2_ for 20 min to reduce endogenous activity and incubated with 10% normal goat serum for 20 min to eliminate nonspecific staining. Next, the primary antibody against BMPRIB (rabbit anti-human polyclonal antibody, Santa Cruz, USA, sc-25455, 1:100) was applied at 4°C overnight. After washing, biotin labeled secondary antibody against rabbit immunoglobulin was applied for 20 min at room temperature. The slides were rinsed and covered with streptavidin-biotin-peroxidase for 20 min. All sections were stained with 3,3′-diaminobenzidinetetra-hydrochloride (DAB). Slides were counterstained with hematoxylin and mounted for light microscopy.

### Evaluation of staining

Using light microscopy, stained tissue sections were reviewed by two pathologists in a blinded manner. A consensus judgment was adopted for the intensity score of the tumors based on the strength of BMPRIB expression. 0 (−): no or low staining; 1 (+): moderate staining; 2 (++): strong staining. Percentage of the positive staining was scored as 0-100. An immunohistochemical score (H score) was ranged from 0 to 200 by multiplying the intensity and the percentage score. Patients were categorized into groups according to H score of BMPRIB: low BMPRIB expression (0-140), high BMPRIB expression (141-200).

Immunohistochemistry for estrogen receptor (ER) and progesterone receptor (PR) was re-evaluated using the 2010 American Society of Clinical Oncology/College of American Pathologists (ASCO/CAP) guideline. Cases were scored positive for ER and PR if nuclear immunoreactivity was present in more than 1% of tumor cells [30]. Immunohistochemistry for human epidermal growth factor receptor 2 (HER2) was re-evaluated using the 2014 ASCO/CAP updated guideline [31].

### Western blot

Western blot analysis was performed to evaluate the expression of BMPRIB in both non-neoplastic breast tissues adjacent to tumor and breast tumor tissues. Frozen breast tumor specimens (13 cases) and non-neoplastic breast tissues adjacent to tumor (13 cases) were collected between 2012 and 2015. All patients were women without preoperative chemotherapy or radiation. Tissues were lysed in 1×SDS lysis buffer (Tris-HCl, pH 6.8, 62.5 mM, 2% SDS, 10% glycerol) for 60 minutes on ice directly. Equal amounts of protein were separated by SDS-PAGE, and electrotransferred onto nitrocellulose membranes. Blots were analyzed by LiCor Odyssey infrared imaging.

### Statistical methods

The SPSS 13.0 software package (SPSS, Chicago, IL, USA) was used for statistical analysis. Mann-Whitney U test and χ^2^ test were performed for group comparisons and correlations between two variables were evaluated by Spearman rank correlation test. The relation between BMPRIB expression and distant metastasis was evaluated using Spearman rank correlation test. Progression-free survival (PFS) was defined as the time from surgery to recurrence or cancer-specific death, whichever occurred first. Overall survival (OS) was calculated from pathological diagnosis to the date of last contact or death from breast carcinoma. Survival analyses were performed according to the Kaplan-Meier method. Wilcoxon matched pairs signed rank test was performed for analyzing the expression of BMPRIB in 32 paired specimens. All statistical tests were 2-tailed and *P* < 0.05 was regarded as significant.

## SUPPLEMENTARY TABLE


