# Supplementary material for: Low expression of BMPRIB indicates poor prognosis of breast cancer and is insensitive to taxane-anthracycline chemotherapy
Source: Oncotarget. 2015 Dec 14;7(4):4770–84. doi: 10.18632/oncotarget.6613 (PMC4826242; doi:10.18632/oncotarget.6613)
Supplement: Supplementary file 1 [file oncotarget-07-4770-s001.pdf]

SUPPLEMENTARY TABLE

Supplementary Table S1: Demographic data of IDC patients

Characteristics	Value
Age	
<50	189
≥50	179
Mean ± SD, Median (range)	51.5± 10.3, 50 (28-89)
Histological grade†	
I	10
II	272
III	64
Estrogen receptor status	
Negative	122
Positive	246
Progesterone receptor status	
Negative	109
Positive	259
HER2⁄neu status	
Negative	291
Positive	77
Events†	
Death	32
Censored	325
Molecular subtypes†	
Luminal A	59
Lumianl B	211
Triple-negative	56
Her2-overexpressing	23

†Some missing data.
IDC: invasive ductal carcinoma
